# Application of Deep Learning Methods in a Moroccan Ophthalmic Center: Analysis and Discussion

**DOI:** 10.3390/diagnostics13101694

**Published:** 2023-05-10

**Authors:** Zineb Farahat, Nabila Zrira, Nissrine Souissi, Safia Benamar, Mohammed Belmekki, Mohamed Nabil Ngote, Kawtar Megdiche

**Affiliations:** 1 LISTD Laboratory, Ecole Nationale Supérieure des Mines de Rabat, Rabat 10000, Morocco; 2 Cheikh Zaïd Foundation Medical Simulation Center, Rabat 10000, Morocco; 3 Cheikh Zaïd Ophthalmic Center, Cheikh Zaïd International University Hospital, Rabat 10000, Morocco; 4 Institut Supérieur d’Ingénierie et Technologies de Santé/Faculté de Médecine Abulcasis, Université Internationale Abulcasis des Sciences de la Santé, Rabat 10000, Morocco

**Keywords:** artificial intelligence, deep learning, diabetic retinopathy, hemorrhages, exudates, automatic screening, method, U-Net, YOLOv5

## Abstract

Diabetic retinopathy (DR) remains one of the world’s frequent eye illnesses, leading to vision loss among working-aged individuals. Hemorrhages and exudates are examples of signs of DR. However, artificial intelligence (AI), particularly deep learning (DL), is poised to impact nearly every aspect of human life and gradually transform medical practice. Insight into the condition of the retina is becoming more accessible thanks to major advancements in diagnostic technology. AI approaches can be used to assess lots of morphological datasets derived from digital images in a rapid and noninvasive manner. Computer-aided diagnosis tools for automatic detection of DR early-stage signs will ease the pressure on clinicians. In this work, we apply two methods to the color fundus images taken on-site at the Cheikh Zaïd Foundation’s Ophthalmic Center in Rabat to detect both exudates and hemorrhages. First, we apply the U-Net method to segment exudates and hemorrhages into red and green colors, respectively. Second, the You Look Only Once Version 5 (YOLOv5) method identifies the presence of hemorrhages and exudates in an image and predicts a probability for each bounding box. The segmentation proposed method obtained a specificity of 85%, a sensitivity of 85%, and a Dice score of 85%. The detection software successfully detected 100% of diabetic retinopathy signs, the expert doctor detected 99% of DR signs, and the resident doctor detected 84%.

## 1. Introduction

Diabetic retinopathy is the primary cause of vision loss in working aged individuals around the world. Its silent progression makes it a sight threatening condition. By 2030, the number of patients with vision-threatening diabetic retinopathy (VTDR) is predicted to jump from 37.3 million to 56.3 million [1]. On the micro scale, according to the Moroccan Society of Ophthalmology [2], the prevalence of DR in Morocco is 35%, approaching 10% of legal blindness. 

Diabetic retinopathy is a small-vessel consequence of diabetes and continues to be the leading cause of preventable ocular morbidity amongst working aged individuals. Diabetes currently affects 422 million people globally, with 600 million people expected to be impacted by 2040, primarily in low- and middle-income nations [3]. It is found in one-third of diabetics and it is related to a higher risk of life-threatening systemic vascular problems such as small vessels heart disease, cardiac failure, and stroke [4]. Prevention is the key factor to reduce the risk of diabetic retinopathy evolution by keeping blood pressure, blood glucose, and blood lipids under control. We distinguish many signs of DR, such as retinal hemorrhages, cottony nodules, and exudates. Diabetic retinopathy is a type of retinal microangiopathy. It entails changes in the vascular wall in addition to modifications in the rheological blood’s characteristics. Although timely laser therapy can help with both macular oedema and proliferative retinopathy, its potential to reverse vision loss is limited. Endo-ocular surgery may be required in rare cases of advanced retinopathy. Common signs and symptoms of Diabetic Retinopathy are blurred vision, sudden onset of double vision, dryness of the eyes, difficulty perceiving colors, floating bodies, and difficulty seeing in the dark.

Using different methods of measurement, several studies have found that the progression of diabetes to diabetic retinopathy (DR) is associated with changes in hemodynamics or measurable vascular geometry. Possible geometric alterations in the retina might indicate the existence of a systemic disease [5]. However, many parameters, mainly venous, showed a significant change in the development of DR, including an early change two years before the start of DR [6].

Furthermore, many studies demonstrated that the genetic polymorphism of histone ethyltransferases, which are responsible for elevated expression of key proinflammatory factors implicated in vascular injury, can be considered as predictors of the risk for micro- and macrovascular diabetic complications [7].

The Diabetes Control and Complications Trial (DCCT) specified that intensive glycaemic control in type 1 diabetes minimized the risk of development of diabetic retinopathy (primary prevention) and slowed its progression in a group with mild retinopathy at baseline (secondary prevention) [8].

Early detection of DR has demonstrated a significant decrease in the risk of vision impairment. Screening programs are conducted within the framework of a healthcare policy for blindness prevention [9]. In Morocco, very few caravans are organized to alleviate the burden on the health system. Nevertheless, these efforts are diluted by the ongoing need for diagnosis, treatment, and further monitoring. 

Other human and geographic challenges are encountered linked to the shortage of trained ophthalmologists and retinal specialists (a ratio of ophthalmologists per capita is 1/68,000), as well as the presence of secluded regions with poor access to medical facilities, as well as uneven scattering between large cities and the countryside [10]. 

In this context, telemedicine and artificial intelligence are on the rise to respond to the pressing demand in healthcare, increasing productivity and efficiency in care delivery. These methods have the ability to be used in every aspect of medical practice, thanks to recent breakthroughs in digital data acquisition. They are moving into fields that were previously regarded as being entirely the domain of humans, and ophthalmology is no exception [3]. Every year, medical caravans visit many remote areas of Morocco to treat patients. Due to the huge inequity between the number of ophthalmologists and the growing number of diabetic populations, we seek to emphasize a feasible solution in this paper, which is relevant to circumstances where computers can be of a great help to health practitioners. We assume that an intelligent system capable of automatically detecting fundus irregularities and DR signs would be beneficial. It could meet this need and pave the way for a true national DR screening program in the future. In summary, our contributions are five-fold:

1. We collected 1000 retinography images from the Cheikh Zaïd Foundation’s Eye Care Center in Rabat and annotated them;
2. We carried out diabetic retinopathy segmentation using U-Net;
3. We carried out diabetic retinopathy detection using Yolov5;
4. We compared the advantages and weaknesses of segmentation and detection methods.
5. We created a software that automatically detects diabetic retinopathy signs.

The remainder of the paper is laid out as follows. Section 2 presents an overview of the existing ophthalmic artificial intelligence solutions. Section 3 is devoted to methods and the dataset. Section 4 deals with experiments and results. Discussion is reported in Section 5. Finally, Section 6 depicts conclusions.

## 2. Related Work

Using the IDRiD dataset [11], Xu et al. [12] worked on a segmentation model called FFU-Net (Feature Fusion U-Net), which improves U-Net architecture. To begin, the network’s pooling layer is replaced with a convolutional layer to minimize the spatial loss of the retinal image. Then, they integrated multiscale feature fusion (MSFF) blocks into the encoders, one that helps the network learn multiscale features and enriches the information provided with skip connections and lower resolution decoders by fusing contextual channel attention (CCA) models. At last, the authors proposed a balanced focal loss function to address misclassification and data imbalance issues. 

Kou et al. [13] proposed an enhanced residual U-Net (ERU-Net) for segmenting microaneurysms (MAs) and exudates (EXs). They evaluated ERU-NET’s performance for MAs and EXs segmentation on three public datasets: IDRiD, DDR, and E-Ophtha. On these three datasets, the used architecture achieves AUC values of 0.9956, 0.9962, 0.9801, 0.9866, 0.9679, and 0.9609 for microaneurysm and exudates segmentation, which are higher than the original U-Net values. 

Li et al. [14] presented MAU-Net, which is a retinal image segmentation method based on the U-Net structure, to segment retinal blood vessels. The authors used DRIVE, STARE, and CHASEDB1 to validate their method. 

Zhang et al. [15] proposed a CNN architecture that incorporated the Inception-Res module, as well as densely connected convolutional modules into the U-Net model. The author tested their model on vessel segmentation from retinal images, MRI brain neoplasm segmentation datasets from MICCAI BraTS 2017, and lung CT scan segmentation data from Kaggle datasets. The results of the lung segmentation achieved an average Dice score of 0.9857. The results for brain tumor segmentation achieved a Dice score of 0.9867. The results for vessel segmentation achieved an average Dice score of 0.9582.

Dai et al. [16] developed a DL solution called DeepDR, which allows users to detect different stages of DR. An amount of 466,247 color fundus images were used for training. The detection results of different DR signs, such as microaneurysms, cotton spots, hard exudates, and hemorrhages were 0.901, 0.941, 0.954, and 0.967, respectively. DR classification into mild, moderate, severe, and proliferative achieved an area under the curves of 0.943, 0.955, 0.960, and 0.972, respectively. 

Sambya et al. [17] used a U-NET model based on a residual network with sub-pixel convolution initialized to the nearest convolution. The suggested architecture was trained and validated on two publicly accessible datasets, IDRiD and e-ophtha, for microaneurysm and hard exudate segmentation. On the IDRiD dataset, the network obtains a Dice score of 0.9998, as well as 99.88% accuracy, 99.85% sensitivity, 99.95% specificity, for microaneurysms and exudates.

Yaday et al. [18] proposed a U-Net based approach for retinal vessel segmentation. Before starting the segmentation procedure, some preprocessing approaches are used to improve the image’s impacted region. Then, a discrete double-tree Ridgelet transform (DT-DRT) is applied to the dataset to extract the features of the region of interest. The proposed segmentation achieved an accuracy of 96.01% in CHASE DB1, 97.65% in DRIVE, and 98.61% in STARE.

Toufique A. Soomro et al. [19] first used preprocessing steps to make the training process more efficient. They implemented the CNN model based on a variational autoencoder (VAE), which is a modified version of U-Net. Their main contribution to the CNN implementation is to replace all pooling layers with progressive convolution and deeper layers. The proposed model generates segmented vessels image. The authors used both DRIVE and STARE datasets to train and test their model. It gave a sensitivity of 0.739 and an accuracy of 0.948 on the DRIVE database. Additionally, a sensitivity of 0.748 and an accuracy of 0.947 are observed for the STARE database.

Swarup et al. [20] compared different architectures (UNET, TLCNN, PCNSVM, and rSVMbCNN). Then, they presented the selected retinal image segmentation method based on a Ranking Support Vector Machine (rSVM) with a convolutional neural network in deep learning for the detection of diabetic retinopathy. They started by computing the pixel-by-pixel score with rSVM, and they then designed a deep convolutional neural network for retinal image segmentation followed by automatic anomaly detection using morphological operations. As a result, they achieved a segmentation accuracy of 96.4%, 97%, and 98.2% for three different databases—STARE, DIARETDB0, and DIARETDB1. 

Many AI devices have been developed to revolutionize DR screening [21]. Pal et al. [22] applied the You Only Look Once version 3 (YOLOv3) algorithm to automatically detect hemorrhages in fundus images. The YOLOv3 algorithm recognized all red spots and surrounded them with multiple boxes. It identifies bounding boxes using a CNN-based model named Darknet53 and a squared error loss function, as well as logistic regression to determine an object’s confidence score. Finally, non-max suppression was used to delete anything other than the best-fit bounding boxes. In order to train their model, the authors used the MESSIDOR dataset. It is made up of 1200 RGB Fundus images. Only 742 people were chosen out of a total of 1200, with 572 going through training and 170 going through validation. The average precision value of test data was 83.3%. 

The results of a YOLO model were published by Rohan Akut [23]. It entails detecting microaneurysms and identifying their location on retinal images. He developed this algorithm based on 85,000 fundus images used for training. The dataset was divided into a ratio of 90–10%, with 90% going to training and 10% going to testing. An amount of 10% of the training dataset was used for validation. The model used enables the creation of a green bounding box around each microaneurysm. 

Ming et al. [24] evaluated EyeWisdom in real world, which is an AI solution based on the YOLO detection system. It was created using 25,297 retinography (3785 from Peking Union Medical College Hospital and Henan Eye Hospital and 21,512 from the Kaggle dataset) [25]. The sensitivity was 90.4%, and the specificity was 95.2%. 

Yang et al. [26] presented a collaborative learning framework for robust DR grading that integrates patch level lesion and image level grading features (CLPI). They used the IDRiD dataset as a lesion dataset, which contains 81 color fundus images (54 for training and 27 for testing) with pixel level annotations of lesions such as exudates, MAs, and hemorrhages. The authors also used image level datasets such as Messidor-1 [27], Messidor-2, LIQ-EyePACs [28], and other private datasets. They demonstrated that the proposed CLPI outperforms senior ophthalmologists, as well as SOTA algorithms. The authors demonstrated the reliability of CLPI by evaluating the DR grading methods in real world scenarios. The findings demonstrated the effectiveness of the lesion attention scheme, as well as the benefits of CLPI’s end-to-end collaborative learning.

Table 1 below summarizes the results of the literature review.

The novelty of this paper lies in creating new software that can automatically detect all types of hemorrhages and exudates. Furthermore, the developed software can recognize laser marks, which are the results of a specific retina therapy and are not pathological signs and differentiate them from both hemorrhages and exudates even if they look similar.

## 3. Methods and Dataset

### 3.1. Methods

#### 3.1.1. Diabetic Retinopathy Segmentation

**Figure 1:**
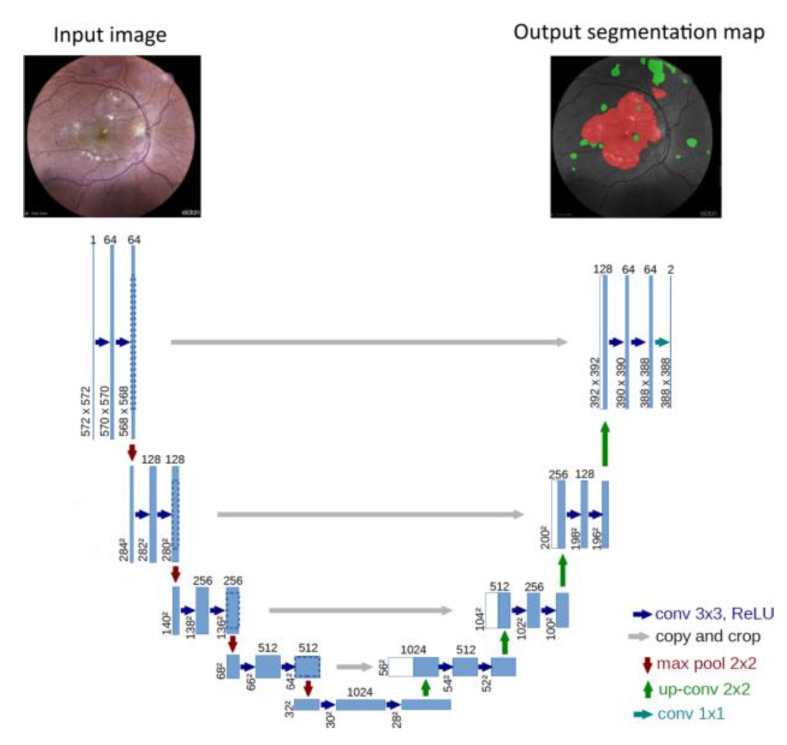
Retinal fundus images with DR signs.

In computer vision and deep learning, image segmentation is the process of partitioning an image into segments or highlighted groups of pixels that are considered as meaningful entities [29]. To segment our color fundus images, we trained and tested a U-Net multi-class segmentation model on 200 labeled retinal images (Figure 1). U-Net [30] has become a widely known medical image segmentation technique and has demonstrated excellent performance as a fully convolutional neural network [3,30]. The U-Net architecture contains two “paths.” First, the contraction path, known as the encoder, is used to capture an image’s context. In fact, it is a combination of convolution and ”max pooling” that not only reduces the image size, but it also generates a feature map, thereby decreasing the number of network parameters. The symmetric expansion path, also known as the decoder, is the second path. Due to the transposed convolution, it also provides precise localization. What characterizes the U-Net architecture is the shortened connections between the layers of equal resolution from the analysis path to the expansion path. These connections provide important high-resolution features for the deconvolution layers.

#### 3.1.2. Diabetic Retinopathy Detection

**Figure 2:**
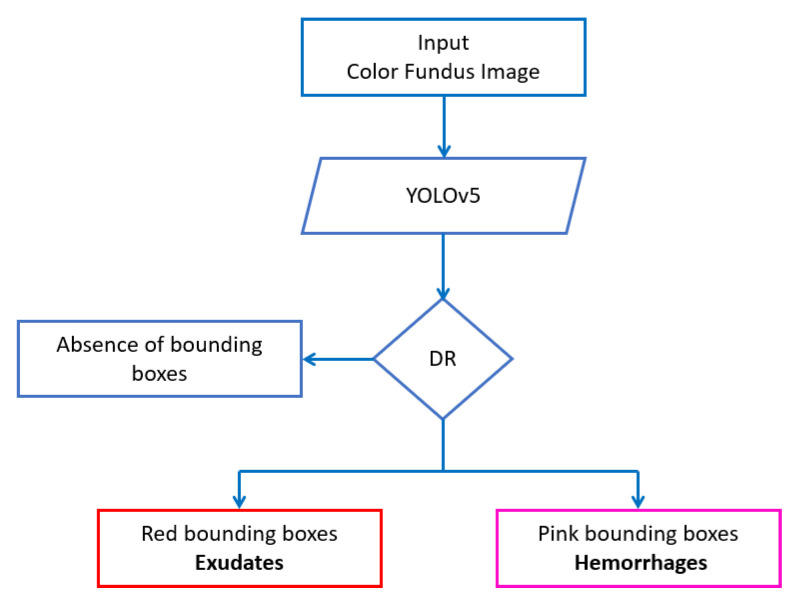
YOLOv5 DR detection.

Object detection is an important branch of both image processing and computer vision. It is the process of detecting occurrences of a particular type of object in videos and images [31]. Deep learning has given a lot of attention to object detection algorithms [32]. The recent YOLO series of algorithms, known for their high speed and precision, have been used in different detection tasks. “YOLO” is an abbreviation for ”You Only Look Once”. In YOLO, a CNN architecture is used only once on the entire image to find the objects. The YOLO system calculates a large number of image features and detects all of the objects. This improves the model’s computational efficiency and makes it more suitable for real-time applications. In our paper, we trained a fifth generation of YOLO model, also known as YOLOv5 architecture, to detect both hemorrhages and exudates (Figure 2). The used model processes the entire image with a single neural network, then divides it into parts and predicts probabilities and bounding boxes for each object. The expected probability is used to weight these bounding boxes. An appropriate selection of the activation function is mandatory for DL networks. Leaky ReLU is used in the middle/hidden layer of YOLOv5, and the sigmoid function is used in the last detection layer [33].

### 3.2. Material

**Figure 3:**
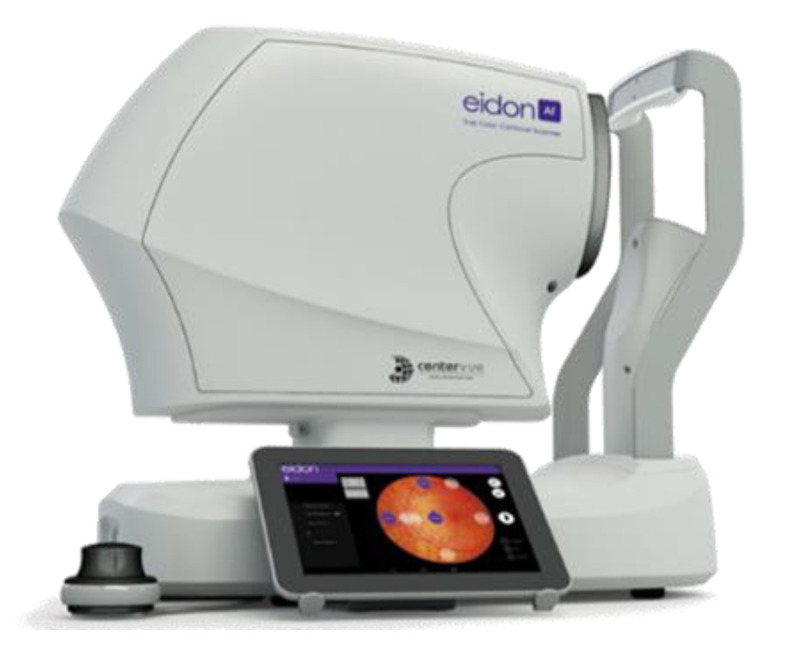
iCare EIDON widefield TrueColor confocal fundus imaging system.

Care EIDON is a non-mydriatic retinal camera (Figure 3). It is one of the first TrueColor confocal systems to set new quality standards in retinal imaging by combining the best features of scanning laser ophthalmoscopy (SLO) systems with those of basic fundus imaging. iCare EIDON is a retinal imaging system that provides high image quality, as well as a confocal view in a non-dilating procedure, as well as a wide field and ultra-high resolution imaging. Furthermore, it distinguishes itself by offering users a variety of imaging modalities, including blue, TrueColor, and red-free and red confocal images, as well as infrared. In addition to this, it enables users to work in both fully manual and fully automated modes and to image through cataract and media opacities. 

Besides, a personal computer is connected to the iCare Eidon in order to facilitate the display, as well as the management and printing of the color fundus images. This computer will be used for the deployment of the proposed software that analyzes images.

### 3.3. Dataset

Our study is approved by the ethics committee of the Cheikh Zaid International University Hospital, and patient consent was obtained.

In order to test both the segmentation and detection methods, 1000 color fundus images were collected from the Foundation Ophthalmic Center of Rabat. These images were taken by the EIDON retinograph, which can automatically produce composite images that allow an overview of the retina of the patient.

#### 3.3.1. Mask Generation for Segmentation

**Figure 4:**
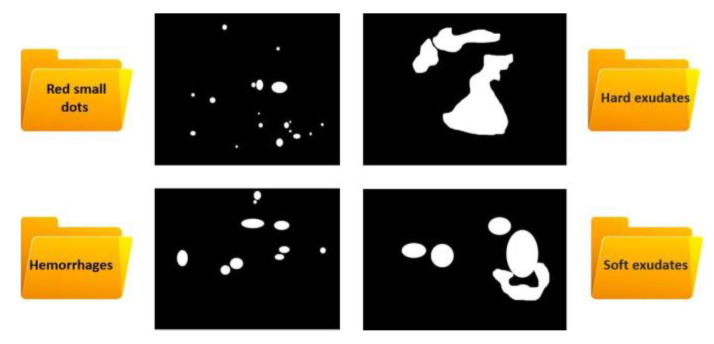
Masks folders of DR signs.

An amount of 200 color images were taken from the Cheikh Zaïd International University in Rabat and then used for training and testing the U-Net algorithm. The image implementation procedure is still in progress. More masks are being manually created in order to improve the model dataset. Gimp software has been used to create masks. After being validated by expert ophthalmologists, all of them were saved as JPEG folders and divided into four folders (hard exudates, soft exudates, hemorrhages, and red small dots) (Figure 4). These four folders were then merged into two folders to keep only two classes, which are hemorrhages (small red dots and hemorrhages) and exudates (hard and soft exudates).

#### 3.3.2. Annotation Generation for Detection

For image detection, a dataset of 1000 local color fundus images taken from the Cheikh Zaïd International University Ophthalmic Center has been used following the approval of the Cheikh Zaïd International University hospital ethics committee. A population with an age range of 39 to 75, a rate of severe pre-proliferative DR of 12%, a rate of complicated DR of 1%, and a rate of legal blindness of 3% was selected. An amount of 70% of our images went for training, 20% went for testing, and 10% went for validation.

**Figure 5:**
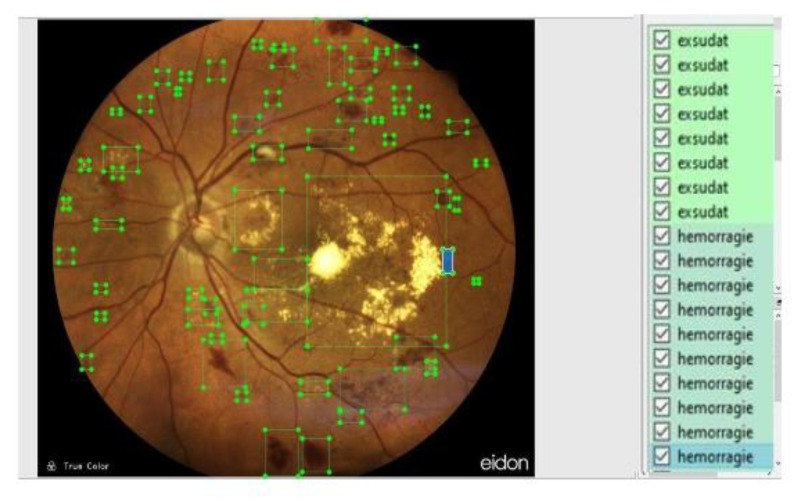
Annotation generation using LabelImg.

Annotations were created manually using LabelImg (Figure 5). Bounding boxes were drawn manually around both hemorrhages and exudates, and then they were saved as ”.txt” files.

**Figure 6:**
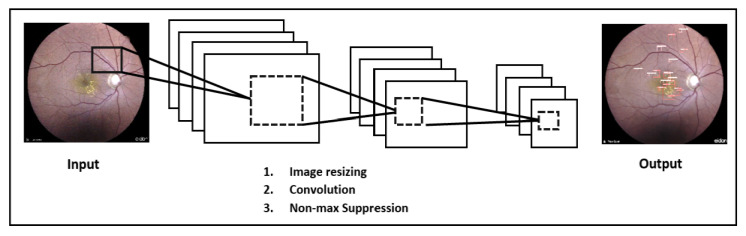
CNN architecture used for detection.

The CNN model starts by resizing the input images to 448 × 448. A convolution is then performed through the horizontal and vertical application of several filters, with the aim of extracting the image features. Finally, to improve the accuracy of the bounding boxes of the detected signs, a removal of the non-max is performed (Figure 6).

## 4. Experiments and Results

We run our notebook on a Colab Pro GPU Tesla P100-PCIE-16GB with high RAM. We also used Pytorch for YOLOv5 and Keras with Tensorflow as the backend for U-Net.

### 4.1. Diabetic Retinopathy Segmentation

**Figure 7:**
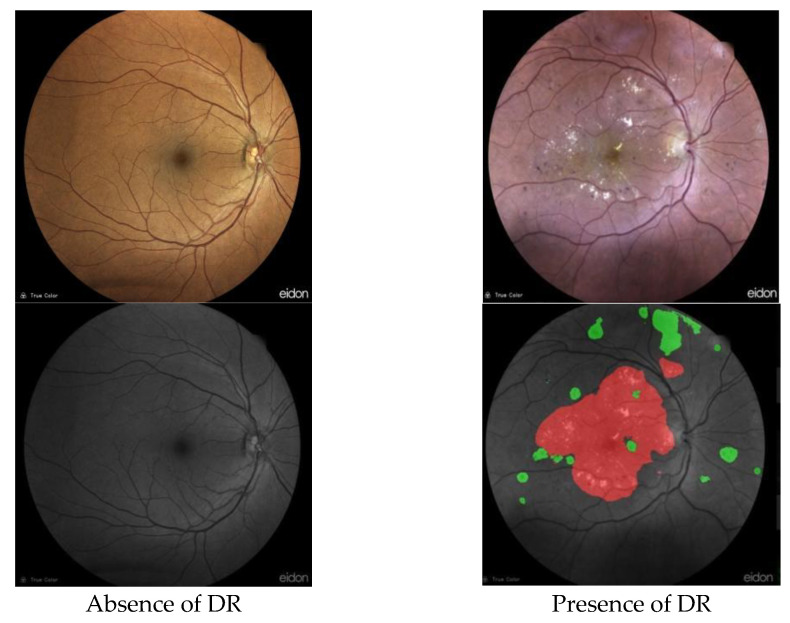
Segmentation results using U-Net.

As preliminary results of the solution under development by our research team, we obtained an output of either the absence or the presence of DR. As shown in Figure 7, the segmentation enables separating DR signs from the retinal image background to make them recognizable by highlighting exudates in red and hemorrhages in green. The use of this technique, however, necessitates the manual creation of training and validation labels.

To assess the adequacy of the proposed solution, evaluation metrics (Table 2) were calculated as follows:

$$J(A,B)\;=\;\frac{|A\cup B-|A\cap B|}{|A\cup B|}$$

*Sensitivity = (True Positive)/(True Positive + False Negative)*

*Specificity = (True Negative)/(True Negative + False Positive)*

$$\mathrm{Dice}=\frac{2\times True\;Positive}{2\times True\;Positive+False\;Negative+False\;Positive}$$

$$\mathrm{IOU}=\frac{A\cap B}{A\cup B}$$

The proposed segmentation method obtained specificity of 85%, a sensitivity of 85%, a Dice score of 85%, and a Jaccard score of 66% for hemorrhages and exudates segmentation.

### 4.2. Diabetic Retinopathy Detection

The fifth version of YOLO was used to detect hemorrhages and exudates in color fundus images. Ultralytics introduced it in June 2020, and it is now known as an advanced object detection algorithm. YOLOv5 provides four versions of the object detection network: YOLOv5s, YOLOv5m, YOLOv5l, and YOLOv5x. In this work, the YOLOv5s model was used. In the YOLOv5 series, YOLOv5s is the network with the smallest depth and width. The following are the specifics of training the YOLOv5 model.

Image size: 640;

Batch size: 16;

Data description: coco128.yaml;

Yolo model: YOLOv5s.yaml;

Configuration (cfg): models are described in YAML model configuration files in “model” directory. There are four versions of models of different sizes. “YOLOv5s.yaml” has been used to train.

**Figure 8:**
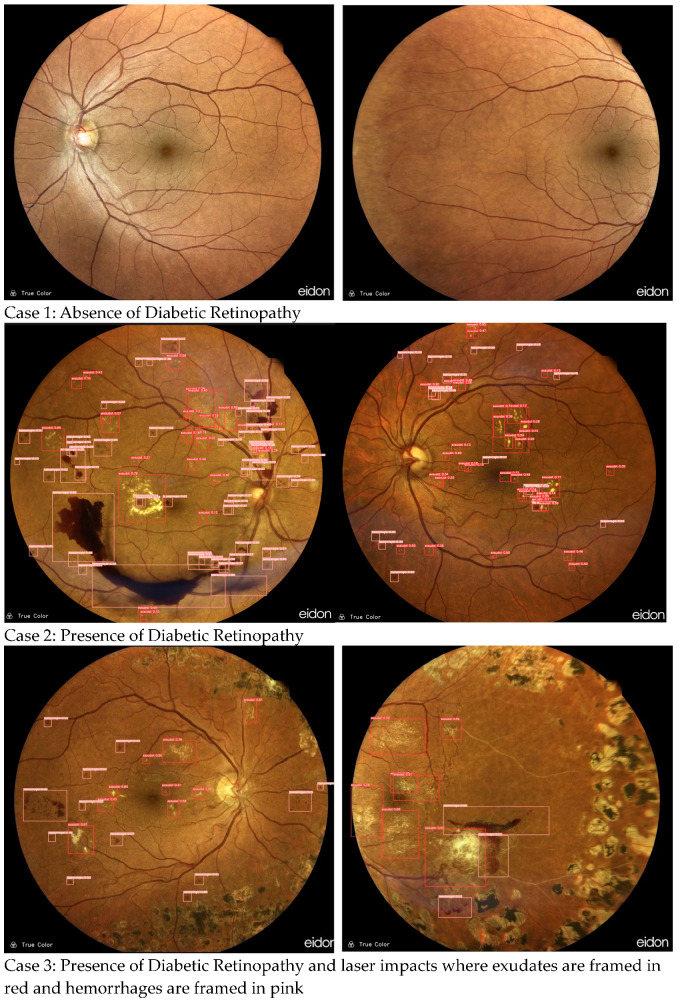
Detection results using YOLOv5.

There are four versions of the model with different sizes. “YOLOv5s.yaml” has been used to train. YOLOv5 was provided with two optimizers: Adam and Stochastic Gradient Descent (SGD). The default one is SGD. For this paper, we used the Adam optimizer, which is a better choice for smaller datasets. After being trained, our detection model allowed us to show diabetic retinopathy signs by drawing red bounding boxes around exudates and pink bounding boxes around hemorrhages. However, in order to assess the detection model’s performance in real-life situations, we tested it on recent color fundus images in order to extend the model in real-world conditions. As depicted in Figure 8, our model was able to differentiate between laser impacts and exudates that look alike.

### 4.3. Abulcasis DR-AI Detection Software

In order to facilitate the use of the detection code, a graphical interface was created using QT Python and FPDF Python.

**Figure 9:**
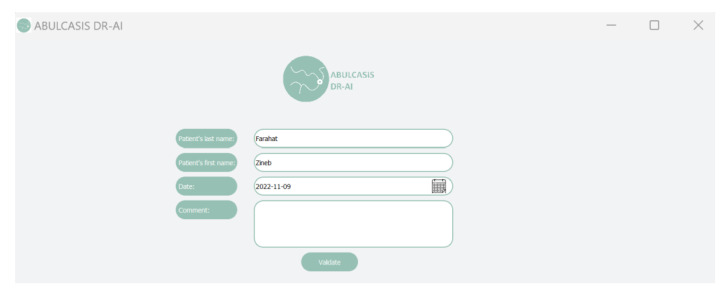
Login interface.

The first interface allows users to fill in fields with different information, such as patient’s name and date, and they can also add comments, as shown in Figure 9. 

A “Validate” button allows the user to validate the filled fields and then to choose the color fundus image path, as well as to switch to the second interface.

**Figure 10:**
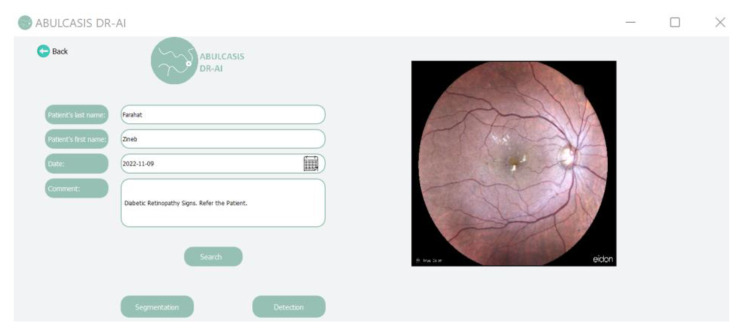
Image choice interface.

The second interface permits us to select a specific image to analyze (Figure 10). Once the “Detection” button is pressed, the third interface is displayed. A “Back” button allows one to go back to the first interface.

The detection result is shown after that, and the user can also enlarge the resulting image in order to see different details of the detected signs of DR.

**Figure 11:**
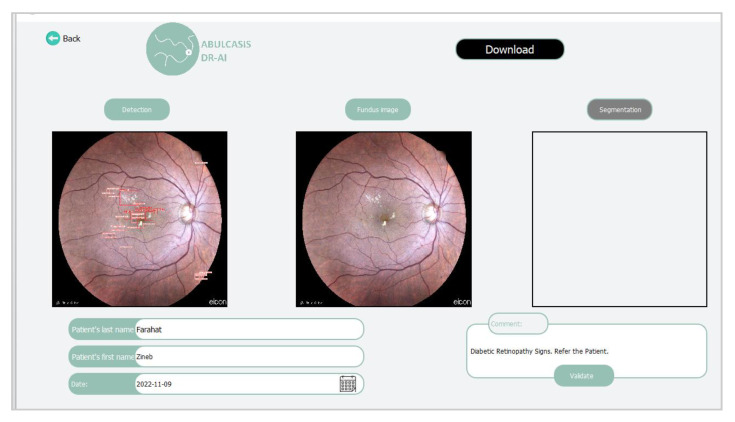
Detection result interface.

Furthermore, it is possible to add comments as shown on Figure 11, so as to describe the patient’s case.

**Figure 12:**
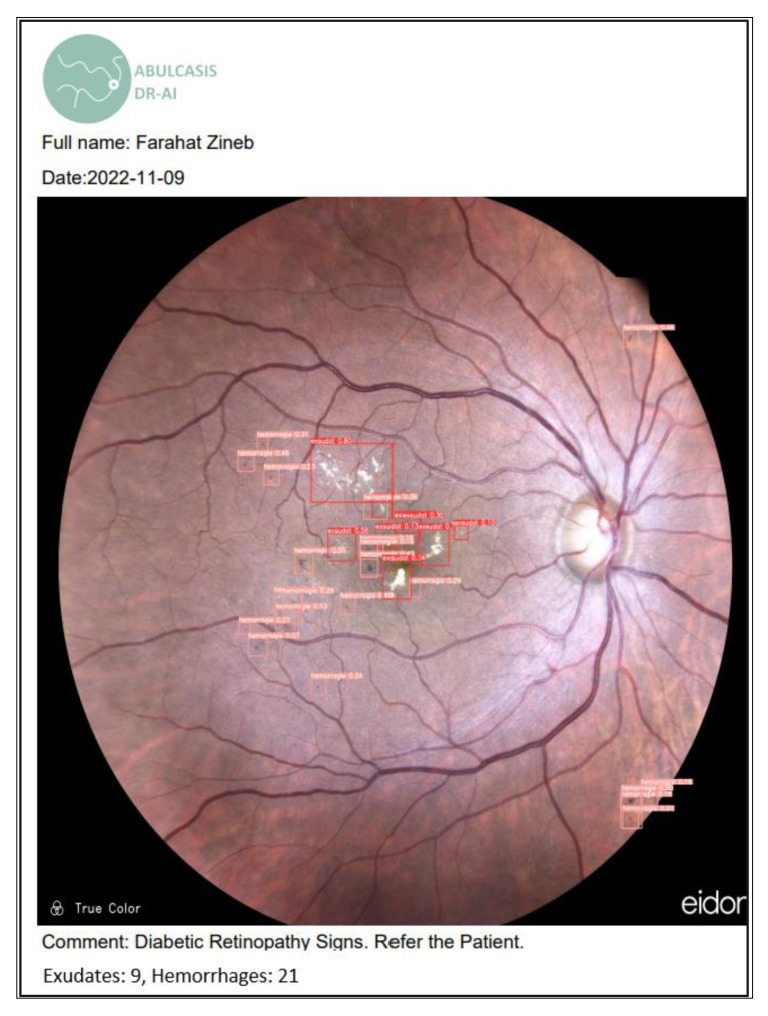
Detection full report.

By clicking on validate a full report is shown containing the implemented information, as well as the resulting color fundus image (Figure 12).

A blind test was performed using different color fundus real images taken directly from the Icare Eidon modality. The Table 3 below shows clinical characteristics of 20 patients.

Comparison results of detection software were summarized and presented in Table 4 below. 

**Figure 13:**
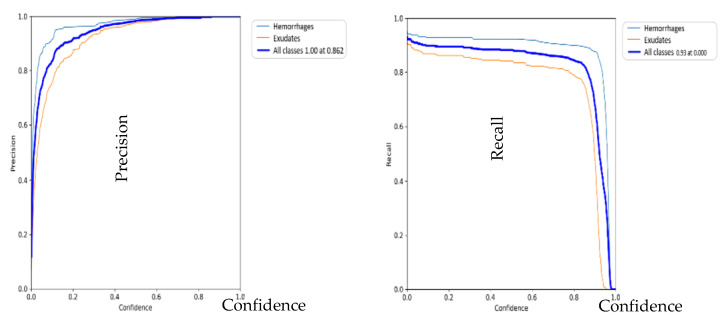
Precision and recall curves.

Figure 13 below represents precision and recall curves obtained using YOLOV5. 

Furthermore, a public dataset named DeepDRID was used in order to test Abulcasis DR-AI software. 

**Figure 14:**
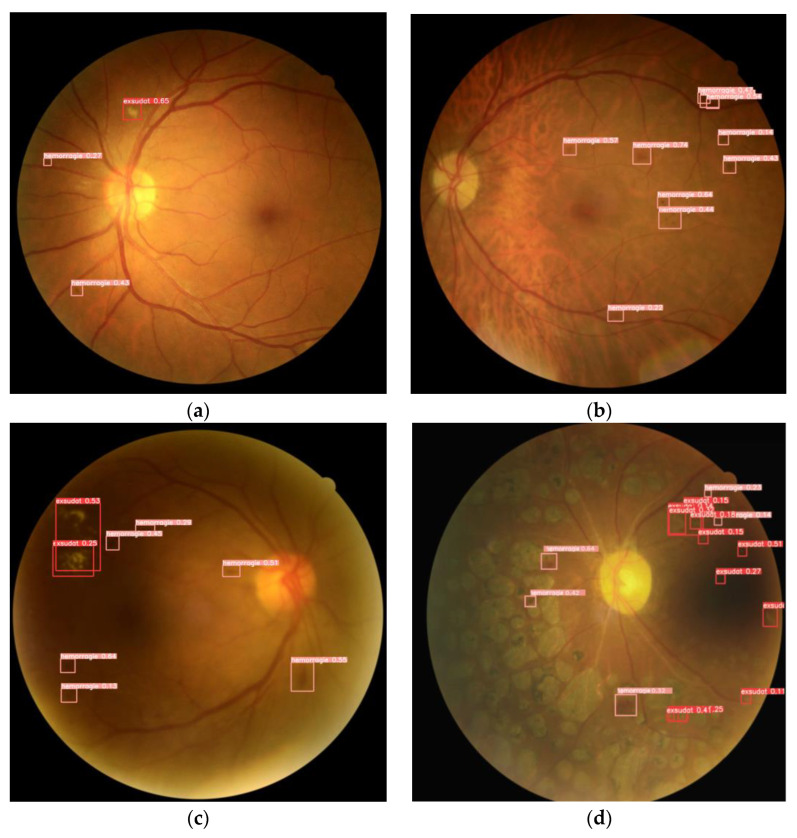
Examples of DR signs detection on DeepDRID public dataset. (**a**) Color fundus image with small hemorrhages and exudates; (**b**) Color fundus image with hemorrhages; (**c**) Color fundus image with hemorrhages and exudates; (**d**) Color fundus image with hemorrhages, exudates and laser impacts.

The proposed software detected all hemorrhages and exudates, as presented in Figure 14. It also recognized laser impacts on the example (d) and did not consider them as pathological signs.

## 5. Discussion

As shown in Table 5, each of the two studied solutions has advantages and limitations. What characterized segmentation is the fact that it allowed us to assign a label to every pixel in our color fundus images. Detection enables users to insert colored bounding boxes around the objects in order to make them recognizable. 

However, since we aim to use this solution for early detection of DR signs, even the smallest exudates and hemorrhages must be taken into consideration and treated to prevent later complications. However, the grey color of the output image may hide unsegmented signs and details. Moreover, the detection method showed better results during the performed blind test.

**Figure 15:**
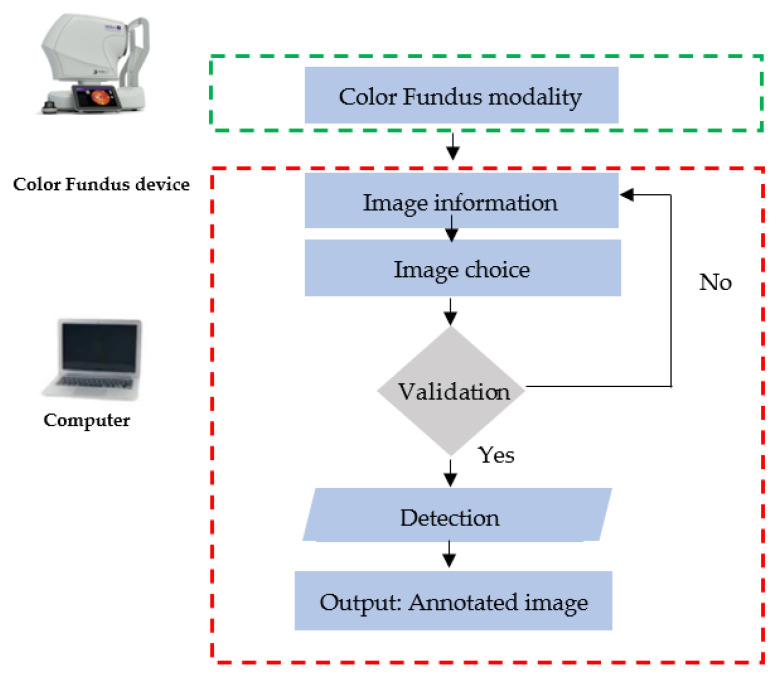
Image detection flowchart.

The proposed software, named Abulcasis DR-AI, allows users to automatically detect DR signs (Figure 15). Differently from the papers presented before, it can detect both types of exudates (hard and soft exudates) and hemorrhages. Abulcasis DR-AI has the capacity to recognize laser impact and does not consider them as DR signs. It has been tested on fundus images, and it has successfully detected 100% of diabetic retinopathy signs, including those not easily detectable with the naked eye. The expert doctor detected 99% of DR signs, and the resident doctor detected 84%. In performing the tests, no false positives were detected. 

Artificial intelligence is the future of early screening of diabetic retinopathy and blindness reduction. The solution we have developed could be used in medical caravans organized in regions with low density of hospital structures, with the aim of optimizing the flow of patients within the hospital structures, and thus it will allow people who do not have access to health care to benefit from medical assistance and to save travel costs for those who do not need an examination by a specialist. 

## 6. Conclusions

With a growing diabetic population and a widening gap between demand and the number of trained resources, we believe that early screening and management of DR must be taken into account. Automation of DR screening would be beneficial in Morocco, where there are few doctors for a fast expanding patient population. Artificial intelligence is the future of early screening to address this health issue and help reduce blindness. The U-Net and YOLOv5 models were used and compared to detect hemorrhages and exudates in retinography images. Therefore, we aim to utilize the proposed method in medical caravans arranged in low-density areas with the goal of improving tele-screening in ophthalmology, optimizing patient flow within healthcare buildings, and allowing people who do not require a specialist checkup to save travel expenses. In upcoming projects, the creation of more labeled retinal images is mandatory for further training of our software in order to improve the metrics and make it able to recognize rare cases of DR. Nevertheless, Abulcasis DR-AI software still has limitations that should be improved, and more investigations should be done in order to create a grading tool of DR severity. 

## Figures and Tables

**Table 1:** Summary of literature review.

Authors	Database	Lesion	Method	Metrics
Xu et al. [8]	Idrid		FFU-Net	SEN = 11.97%, IOU = 10.68%, and DICE = 5.79%
Kou et al. [9]	E-Ophtha, idrid and DDR	Microaneurysms (MAs) and exudates (EXs)	ERU-Net	Idrid: AUC = 0.9956 DDR, and E-Ophtha
Li et al. [10]	DRIVE, STARE, and CHASEDB1	Retinal blood vessels	MAU-Net	DRIVE: ACC = 0.9557 STARE: ACC = 0.9581 CHASEDB1: ACC = 0.9620
Zhang et al. [11]	Kaggle	Retinal blood vessels	U-Net model	Dice score = 0.9582
Dai et al. [12]	Local fundus images	Microaneurysms, cotton spots, hard exudates, and hemorrhages	Deepdr developed based on resnet41 and Mask-RCNN57	0.901, 0.941, 0.954, and 0.967
Sambya et al. [13]	Idrid and e-ophtha	Microaneurysms and hard exudates	U-NET	Dice score = 0.9998, accuracy = 99.88%, sensitivity = 99.85%, specificity = 99.95%
Yaday et al. [14]	CHASE DB1, DRIVE and STARE	Retinal blood vessels	U-NET	-CHASE DB1: Accuracy = 96.01%-DRIVE: Accuracy = 97.65% STARE: Accuracy= 98.61%.
Toufique A. Soomro et al. [15]	DRIVE and STARE	Retinal blood vessels	U-Net	-DRIVE: Accuracy = 0.948 Sensitivity = 0.739 STARE: Accuracy = 0.947 Sensitivity = 0.748
Swarup et al. [16]	STARE, DIARETDB0 and DIARETDB1	Exudates	Ranking Support Vector Machine (rsvm) with a convolutional neural network	Accuracy of 96.4%, 97% and 98.2%
Pal et al. [18]	MESSIDOR	Hemorrhages	YOLO	Precision = 83.3%
Rohan Akut [19]	Eyepacs	Microaneurysms	YOLO	Precision = 86.7%
Yang et al. [22]	Idrid, Messidor-1, Messidor-2, LIQ-eyepacs, and a private datasets	Exudates, MAs, and hemorrhages.	CLPI	MESSIDOR: AUC = 0.946 LIQ-EYEPACS: AUC = 0.916 Private Database: AUC = 0.983

**Table 2:** Segmentation metrics.

Metric	Value
Jaccard	0.6652
Sensitivity	0.8509
Specificity	0.8506
Dice	0.8506
IOU	0.7503

**Table 3:** Clinical characteristics of the patients.

Age	Percentage
20–40	10%
40–60	65%
60–80	25%
**Sex**	
Male	30%
Female	70%
**Diabetic Retinopathy**	
Presence	55%
Absence	45%

**Table 4:** Detection comparison results.

	Expert Doctor	Resident Doctor	Abulcasis DR-AI	Correct Answer
Total Exudates	98.2%	84.4%	100%	100%
Total Hemorrhages	100%	83.6%	100%	100%
Mean Detection	99%	84%	100%	100%

**Table 5:** Advantages and limitations of both segmentation and detection approaches.

Approach	Segmentation	Detection
Advantages	-Highlights the segmented parts of the image with different colors, which makes them easily recognizable. -Highlights the segmented parts of the image with different colors, which makes them easily recognizable. -Detects, classifies, and segments every object by assigning a label to each pixel of the image.	-Inserts a bounding colored box around each object.
Limitations	-Requires the creation of learning masks. -The grey color of the output image may hide unsegmented signs.	-Requires the creation of learning masks.

## Data Availability

A local dataset was created for training and testing the software, nevertheless it is unavailable due to privacy and ethical restrictions. The software was also tested using a public dataset called DeepDRID: github.com/mutual-ai/Deep-Diabetic-Retinopathy-Image-Dataset-DeepDRiD-, accessed on 20 February 2023.
